# Knowledge and Practices of Child Eye Healthcare Among Parents in Aseer Region, Saudi Arabia

**DOI:** 10.7759/cureus.30404

**Published:** 2022-10-17

**Authors:** Waleed Aldhabaan, Ziyad M Alkhammash, Ahmed S Al Zomia, Yazeed Alshahrani, Razan Asiri, Mushari M Alqhtani, Wejdan Alnahdi, Yahya Alqahtani, Sultan Alqahtani, Anas Asiri, Bander Asiri

**Affiliations:** 1 Ophthalmology, King Khalid University, Abha, SAU; 2 Medicine, King Khalid University, Abha, SAU; 3 Medicine and Surgery, King Khalid University, Abha, SAU; 4 Ophthalmology, Aseer Centeral Hospital, Abha, SAU; 5 Surgery, King Khalid University, Abha, SAU; 6 Medicine, King Saud Bin Abdulaziz University for Health Sciences College of Medicine, Jeddah, SAU; 7 Psychiatry, Asir Central Hospital (ACH), Abha, SAU

**Keywords:** visual complications, visual, child blindness, parents’ awareness, children's, eye care

## Abstract

Background: The majority of causes of childhood blindness are preventable and treatable. There are an estimated 1.4 million blind children worldwide, with roughly three-quarters of them living in developing countries. In most low-income countries, school-age children account for 20%-30% of the total population.

Aim: To evaluate parents' knowledge, attitudes, and practices related to pediatric eye medical services in Saudi Arabia's Aseer region.

Methodology: A descriptive cross-sectional approach was used targeting all parents in the Aseer region. Data were collected using a structured questionnaire developed by the study investigators. The questionnaire included parents’ sociodemographic data and a family history of blindness or visual disability. Parents’ awareness regarding pediatric eye care was assessed using relevant items. The parents’ practices and attitudes regarding eye care were also assessed within the questionnaire.

Results: The study included 899 parents who replied to the online questionnaire in its entirety. Some 54% of the responding parents were aged 30-50 years, and 51.2% were males. Of the parents, 46.2% had a university-level education, and 48.5% accompanied their children for eye examinations. About 65% of the parents knew about clinics for eye examinations, and 63.3% of them knew that blind children could learn. In total, more than one-third of the parents were aware of pediatric eye care.

Conclusions and Recommendations: The study found that parents were aware of pediatric eye health and sought eye care for their children. More effort should be put forth through planned awareness programs to educate parents and assist them in overcoming the fears and barriers that keep them from seeking eye care for their children.

## Introduction

Children can experience a multitude of eye conditions, including both anatomical problems in the various structures of the eye and difficulties with their vision [1-3]. To prevent blindness, pediatric eye care comprises a continuum of eye care for children that can include both vision screening and comprehensive eye exams [4]. All children, even those with no signs of ocular morbidity should have their eyes checked at regular intervals. Any child who experiences vision problems or shows symptoms of eye disease should receive a comprehensive eye exam by an eye doctor (optometrist or ophthalmologist) [5].

Limiting childhood blindness is a priority for meeting the goals of Vision 2020, the World Health Organization's (WHO) program for eliminating avoidable blindness [6]. This is a priority because the number of blind years (the number of years a blind person lives after becoming blind) caused by childhood blindness is second only to those caused by cataract, and because 50% of blindness in children is avoidable [7-9].

Coordination at all levels of eye care is required for children's eye care. If avoidable blindness in children is to be prevented -- awareness at the primary, secondary, and tertiary prevention levels is required. Implementation of a highly skilled pediatric eye care team is also essential [10]. Parents, as the main caregivers, are mainly responsible for seeking healthcare services for their children [11]. Mapping parents’ attitudes, practices, and awareness of their children’s eye problems and required health care are essential to the exploration of why some parents take care of their children’s eye health, whereas others do not [12-14]. This understanding is vital for early detection, and intervention is most effective when performed at an early age [15-16]. This current study aimed to assess parents’ awareness and practices regarding children’s eye healthcare in the Aseer region, southern Saudi Arabia.

## Materials and methods

A descriptive, cross-sectional approach targeting all parents in the Aseer region was used. The Ethical Committee of Scientific Research at King Khalid University approved the study with approval number (ECM#2020-158)-( HAPO-06-B-001).

Parents with children aged 5-12 years who had been residing with their children for at least the past year were included in this study. Data were collected using a structured questionnaire developed by the study investigators after an intensive literature review and expert consultation. The data collected on the questionnaire included parents’ sociodemographic characteristics, such as age, gender, education, and marital status. A family history of blindness or visual disability was also included. Parents’ awareness regarding children’s eye care was assessed using 11 questions, including the importance of eye care, the relationship between the eye and visual problems and learning, the history of eye checkups for the child, and knowledge about schools for blind children. The parents’ practices and attitudes regarding eye care were also assessed with the questionnaire. A panel of three experts independently reviewed the questionnaire for content validity, and all suggested changes were implemented until the tool was finalized. The questionnaire was uploaded online via social media platforms by the researchers and their relatives to be completed by all eligible parents. Initial questions asked of eligible parents included whether they had children of the appropriate age and whether they had lived with them in the previous year.

Data analysis

Following extraction, the data were revised, coded, and processed using IBM SPSS version 22 statistical software (IBM Corp., Armonk, NY). Two-tailed tests were utilized in all statistical analyses. A statistically significant result was defined as a P value less than 0.05. For awareness items, each correct answer received one point, and the sum of the discrete scores for the various items was calculated. A patient with less than 60% (6 points) of the maximum score was considered to have poor awareness, while a score of 60% (7 points) or higher was considered to have good awareness. All variables, including demographic data, awareness items, and parental practices and attitudes, were subjected to a descriptive analysis based on frequency and percent distribution. The Pearson chi-square test was used to test for univariate relationships between parents' socio-demographic data and practices and their level of awareness.

## Results

The study included 899 parents who replied to the online questionnaire in its entirety. Exactly 54% of the responding parents were aged 30-50 years, and 51.2% were males. Of the parents, 46.2% reported having a university-level education, and 73.6% were married and living with their families (not separated). About 25% of the parents had blind or visually disabled children in their families, and 74.4% of the causes of blindness were explained by physicians (Table 1).

**Table 1:** Socio-demographic data of respondent parents in the Aseer region.

Socio-demographic data		No	%
Age in years	< 20 years	94	10.5
	20-30	266	29.6
	30-40	274	30.5
	40-50	211	23.5
	>50	54	6.0
Gender	Male	460	51.2
	Female	439	48.8
Educational level	Basic education	243	27.0
	Secondary education	241	26.8
	University education	415	46.2
Marital status	Married	662	73.6
	Divorced|widow	237	26.4
Had blind children in your family	No	581	64.6
	Yes	223	24.8
	Don’t know	95	10.6
If yes, the cause was explained by the physician	No	35	15.7
	Yes	166	74.4
	Don’t know	22	9.9

Regarding parents’ practices, Table 2 shows that 48.5% of the respondent parents accompanied their children to eye examinations, and 46.6% of them did so when the children were younger than school age. Of those who did not accompany their children to eye examinations, 59% asserted that their children could see well, and 47.5% believed that there was no need for an eye examination, while 23.3% did not seek eye examinations for their children due to a lack of money.

**Table 2:** Child eye care practice as recorded by sampled parents in the Aseer region.

Eye care practice		No	%
Accompanied your child for eye examination	No	463	51.5
	Yes	436	48.5
If yes, before going to school (n=436)	No	184	35.9
	Yes	239	46.6
	Don’t Know	90	17.5
Causes of not going (n=463)	Don’t know that my child need eye examination	145	31.3
	No need for eye examination	220	47.5
	Lack of money	108	23.3
	Child see well	273	59.0

Table 3 shows parents’ awareness regarding eye care for their children. About 65% of parents knew about eye examination clinics, and 63.3% of them knew that blind children could learn. Moreover, 60.2% of parents knew that children with visual defects or blindness could go to school, and 59.4% of them thought that blind children could deal with their colleagues. Young children’s liability for visual impairment was recorded by 58.4% of the parents, and only 37.9% of the parents said that a blind child would be annoyed by his or her colleagues. In total, 38.9% of parents had a good level of awareness regarding pediatric eye care.

**Table 3:** Parents’ awareness regarding eye care among children in Aseer region.

Eye care awareness items		No	%
There are clinics for eye examination	No	177	19.7
	Yes	587	65.3
	Don't know	135	15.0
Old age only had eye problems	No	525	58.4
	Yes	264	29.4
	Don't know	110	12.2
Children with visual defect or blind can go to school	No	172	19.1
	Yes	541	60.2
	Don't know	186	20.7
Blind children can learn	No	185	20.6
	Yes	569	63.3
	Don't know	145	16.1
Blind child can deal with colleges	No	161	17.9
	Yes	534	59.4
	Don't know	204	22.7

Blind child will be annoyed from his college	No	252	28.0
	Yes	341	37.9
	Don't know	306	34.0
Know schools for blind children	No	385	42.8
	Yes	346	38.5
	Don't know	168	18.7
Overall awareness	Poor	549	61.1
	Good	350	38.9

**Figure 1:**
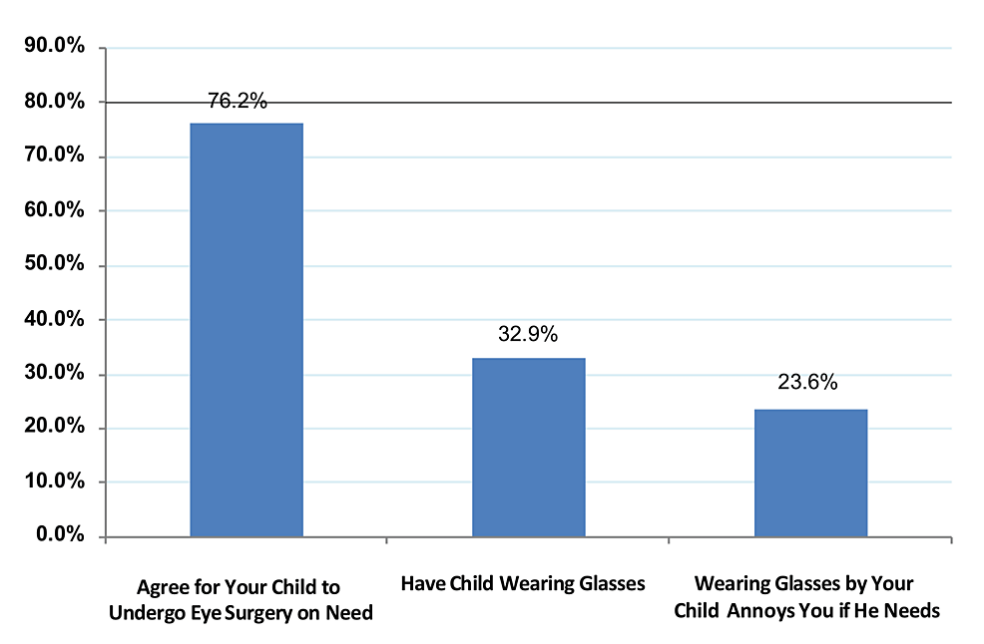
Parents’ attitude regarding eye care among their children.

Figure 1 shows parents’ attitudes toward pediatric eye care. Exactly 76.2% of parents agreed that their children could undergo eye surgery if needed, and 32.9% agreed on their children wearing glasses for a visual defect, while only 23.6% were annoyed if their children wore glasses.

Finally, Table 4 shows the distribution of parents’ awareness regarding pediatric eye care according to their personal data, practices, and attitudes. About 48% of parents aged 50 years and older had a good awareness level compared to 22.3% of parents below the age of 20 years. This difference was found to be statistically significant (p=0.001). High awareness was recorded among 54.7% of university-educated parents compared to 18.1% of parents with a basic education level (p=0.001). Moreover, 43.5% of parents who lived with their families had a good awareness level. About 49% of parents who had a blind child in their family had a good awareness level compared to 39.9% of those who did not (p=0.001). Parents who accompanied their children for eye examinations had a higher awareness level than those who did not (51.8% vs. 26.8%, respectively). Exactly 43.8% of parents with a positive attitude towards their children undergoing eye surgery had a good awareness level compared to 23.4% of those who did not agree with eye surgery (p=0.001).

**Table 4:** Distribution of parents’ awareness regarding children's eye care according to their personal data, practice, and attitude. P, Pearson's X^2^ test; *p < 0.05 (significant)

Factors		Awareness level				p-value
		Poor		Good		
		No	%	No	%	
	< 20 years	73	77.7	21	22.3	0.001*
	20-	174	65.4	92	34.6	
Age in years	30-	145	52.9	129	47.1	
	40-	129	61.1	82	38.9	
	50+	28	51.9	26	48.1	
Gender	Male	274	59.6	186	40.4	0.344
	Female	275	62.6	164	37.4	
Educational level	Basic education	199	81.9	44	18.1	0.001*
	Secondary education	162	67.2	79	32.8	
	University education	188	45.3	227	54.7	
Marital status	Married	374	56.5	288	43.5	0.001*
	Not married	175	73.8	62	26.2	
Had blind children in your family	No	349	60.1	232	39.9	0.001*
	Yes	114	51.1	109	48.9	
	Don't know	86	90.5	9	9.5	
Accompanied your child for eye examination	No	339	73.2	124	26.8	0.001*
	Yes	210	48.2	226	51.8	
Have child wearing glasses	No	443	73.5	160	26.5	0.001*
	Yes	106	35.8	190	64.2	
Agree for your child to undergo eye surgery on need	No	164	76.6	50	23.4	0.001*
	Yes	385	56.2	300	43.8	

## Discussion

Visual impairment and blindness in children in developing countries are typically caused by preventable and treatable conditions [17]. A lack of awareness about preventive eye care measures among parents or guardians and community members, as well as knowledge of where to get appropriate care, is a major contributor to childhood blindness [18]. Every year, approximately 500,000 children become blind, and 1.5 million are already blind. These figures are five times higher in low-income areas compared to high-income areas. A child is estimated to go blind every minute, and 60% of such children die within a year of becoming blind; such is the gravity of the situation. Africa is home to an estimated 1.3 million blind children [18], but the relatively low refractive error (RE) (1.8%) in most African countries is reportedly too low to justify RE screening prioritization [19-20]. Studies on eye disease awareness in developed and developing countries have found that many people seek timely eye care to reduce the burden of blindness, even among children. Poor health literacy leads to poor health-seeking behavior among parents.

The current study aimed to assess parents’ awareness regarding eye healthcare for their children, as well as their practices and attitudes towards pediatric eye examination and the management of visual impairment. The study revealed that the majority of respondent parents were male, in the middle-age group, and not distinguished by a high education level. Some 223 (24.8%) of the responding parents had blind children in their families. Parents’ awareness regarding pediatric eye care was moderately poor, as only two parents out of every five had a good awareness level. The highest areas of awareness were parents’ knowledge about eye examination clinics and the ability of blind children to attend school and learn, while awareness was lacking regarding the relationship between blind children and their colleagues. The factors most closely associated with a high level of awareness included maturity (age above 40 years), a higher education level, having blind children in their families, which motivated them to have their children examined to avoid this problem, a positive attitude, and a good practice level.

As for parents’ practices regarding pediatric eye healthcare, some 436 (48.5%) of the respondent parents reported accompanying their children to undergo an eye examination. Among those who did not, the main barrier was their perception that an eye examination was not needed, followed by their perception that the child could see well enough, and a lack of money was the main reason given by 108 (23.3%) of them. As for their attitudes, more than 76.2% of the parents agreed that their children could undergo surgery if needed, and only 23.6% reported that they would be upset if their children wearing glasses.

A study was conducted in Nigeria to assess the factors that influence parents to seek eye care for their children [21]. The study revealed that parents were more likely to seek care for symptomatic visual problems than for conditions they could not perceive. A family history of ocular disease and repeated complaints about visual problems motivated parents to seek eye care for their children. The cost of eye‐care services was a major barrier. In India, a study was conducted to assess the awareness regarding common childhood ocular problems among parents visiting a pediatric Outpatient Department (OPD) in a tertiary-level hospital in the State of Uttarakhand [22]. Awareness regarding various causes of childhood blindness among parents included in the study ranged from 19% to 89%. Conditions that could lead to childhood blindness were significantly related to their place of residence and education level. Urban parents were more aware of the risks associated with not wearing spectacles regularly.

More than 90% of parents knew that childhood blindness could be avoided, and 92% knew it could be treated. However, only 30% of parents took their children for regular eye exams. The lack of programs in communities and schools to identify children who need examination, treatment, referral, or rehabilitation adds to the financial burden on parents, guardians, and caregivers.

Limitations

The current study contains a number of limitations. First, despite our efforts to assure the clarity of the questionnaire items, a major limitation of our study is the reporting bias and incorrect interpretation of several questions. The fact that we conducted this research in Saudi Arabia's Aseer region may have impacted its generalizability. Due to the small number of participants, it will be difficult to evaluate parents' practices, attitudes, and knowledge of pediatric eye health in the Aseer region.

## Conclusions

In conclusion, the study revealed that parents had some awareness and sought care for their children’s eye health. Awareness and practice were mainly associated with having a history of exposure to a blind child or similar cases in the family. Researchers suggest that more effort be put forth through planned awareness programs to educate parents and assist them in overcoming the concerns and barriers that prevent them from seeking eye healthcare for their children.
